# Advances and opportunities in gene editing and gene regulation technology for *Yarrowia lipolytica*

**DOI:** 10.1186/s12934-019-1259-x

**Published:** 2019-11-29

**Authors:** Vijaydev Ganesan, Michael Spagnuolo, Ayushi Agrawal, Spencer Smith, Difeng Gao, Mark Blenner

**Affiliations:** Department of Chemical and Biomolecular Engineering, Clemson University, 206 S. Palmetto Blvd., Clemson, SC 29634 USA

**Keywords:** *Yarrowia lipolytica*, Metabolic engineering, Synthetic biology, Genetic tools, Genome editing, CRISPR–Cas9, Transposon, Functional genomics

## Abstract

*Yarrowia lipolytica* has emerged as a biomanufacturing platform for a variety of industrial applications. It has been demonstrated to be a robust cell factory for the production of renewable chemicals and enzymes for fuel, feed, oleochemical, nutraceutical and pharmaceutical applications. Metabolic engineering of this non-conventional yeast started through conventional molecular genetic engineering tools; however, recent advances in gene/genome editing systems, such as CRISPR–Cas9, transposons, and TALENs, has greatly expanded the applications of synthetic biology, metabolic engineering and functional genomics of *Y. lipolytica*. In this review we summarize the work to develop these tools and their demonstrated uses in engineering *Y. lipolytica*, discuss important subtleties and challenges to using these tools, and give our perspective on important gaps in gene/genome editing tools in *Y. lipolytica*.

## Background

*Yarrowia lipolytica* is a non-conventional yeast that has been used as a safe and robust host to produce single cell proteins [1], lipids [2], and organic acids [3] at an industrial scale. Its oleaginous behavior means that it can naturally accumulate significant amounts of neutral lipids (> 20% w/w) under nutrient limiting condition [4]. With advances in understanding of its de novo lipogenesis and efforts towards rewiring native metabolic pathways for lipid accumulation, engineered strains can produce up to 90% of their dry cell mass as lipids, and achieve lipid productivity as high as 1.2 g/h/L with enhanced maximum theoretical lipid yield [5–7]. These extensive engineering efforts have made *Y. lipolytica* an attractive biomanufacturing platform for industrial production of lipid-derived chemicals and fuels.

Without additional engineering, *Y. lipolytica* can efficiently utilize several C6 sugars including glucose, fructose and mannose, hydrophobic substrates such as lipids and alkanes, glycerol, and acetate as carbon sources [8–11]; however, it is unable to grow on some of the most abundant and inexpensive substrates, such as carbon dioxide, xylose, and lignocellulose. Over the past several years, *Y. lipolytica* has been engineered to improve the range of substrates that can be utilized for growth and production [10, 12, 13]. By expressing some combination of transporters, enzymes for lignocellulosic hydrolysis, or enzymes for metabolism of novel substrates, the engineered strains can grow efficiently on xylose [13–17], galactose [18], cellobiose [19, 20], sucrose [3, 21], and polysaccharides such as starch [22], cellulose [23], and xylan [24]. These engineering efforts have facilitated economical production of value-added products from renewable feedstocks. Similarly, *Y. lipolytica* has been engineered to produce a variety of non-inherent compounds including polyunsaturated fatty acids [2, 25], terpenoids [26], carotenoids [27–31], diacids [32, 33], alcohols [34–36], and polyketides [37]. Numerous comprehensive reviews supporting the strength of *Y. lipolytica* over other yeast for industrial production are already available elsewhere [10, 12, 13, 38–44].

All of these efforts have been possible thanks to the advances in our understanding of the *Y. lipolytica* metabolic network, molecular genetics, and the continued development of genetic tools for engineering *Y. lipolytica*. There are several excellent reviews on the basic genetic engineering tools, such as host strains and markers, vectors, promoters, terminators and replication elements [45–50]. To date, there is one review on the application of CRISPR–Cas9 for metabolic engineering of *Y. lipolytica* from 2018 [51]. Here, we focus this review on the development of gene and genome editing systems, and their applications in functional genomics and metabolic engineering, which are summarized in Table 1. We have included the most recent advances that were not in the literature when the Shi review was written. We also discuss the subtleties and challenges of using these tools, and we provide our perspective on the future of gene and genome editing tools that will be needed to exploit the full potential of this non-conventional yeast.

**Table 1:** Summary of all gene and genome editing reports in *Y. lipolytica*

Tool	Notes	Addgene	Refs
Cas9 indel and integration	Hybrid promoters combining strong RNA pol II promoters and tRNA lead to high efficiency indel	70007	[51]
Cas9 indels, multiplexed	Multiplexed indels by multiple sgRNAs processed with hammerhead ribozymes	73226	[52]
Cas9 indels	T7 polymerase expression of sgRNA; Addition of GGG improved sgRNA activity	N/A	[53]
Cas9 integration	Non-essential landing sites were established for markerless integration	84608-17	[27]
Cas9 deletion and integration	Two sgRNAs used to excise DNA regions. Simultaneous integration by HMEJ	N/A	[55]
Cas9 integration	EasyCloneYALI: integration of non-replicating constructs by HR at 11 landing sites in Ku70 knockout	100000 0140-141	[56]
TALENs indel and integration	TALEN induced DSB can create indels or mediate HR based integration	N/A	[57]
dCas9-MxiI	sgRNA targeted upstream of the TSS	91248	[58]
dCas9 dCpfI	Multiplexed targeting of both dCas9 and dCpfI a pathway	N/A	[59]
CRISPR-VPR	CRISPRa upregulated two genes for cellobiose metabolism	N/A	[60]
PiggyBac	TTAA specific transposon insertion; excision/integration mutant used for recycle selection markers	N/A	[66]
Hermes Tn	Sequence independent transposon insertion; useful for functional genomics and strain engineering	113332	[67]
Cas9 genome scale indel	Sixfold redundant sgRNA for entire genome; useful for functional genomics and strain engineering	N/A	[68]

## Gene and genome engineering tools for *Y. lipolytica*

**Fig. 1:**
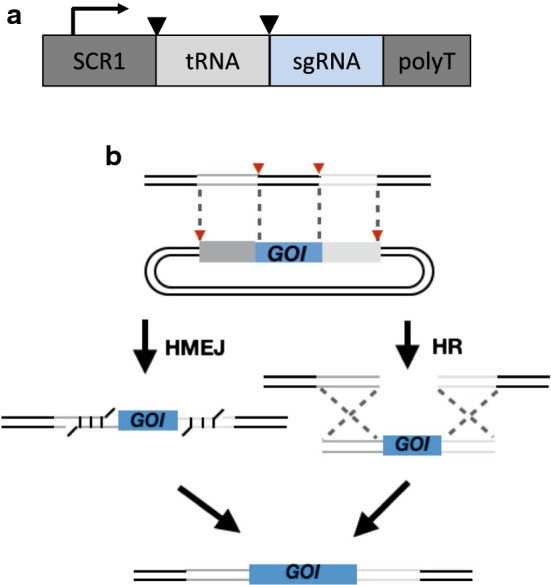
CRISPR–Cas9 innovations. **a** Hybrid promoters of RNA polymerase III promoters and tRNA lead to efficient sgRNA expression and processing. **b** Inclusion of gRNA target sites on the transgene plasmid lead to two possible site specific modes of integration—homologous recombination (HR) and HR-independent homology mediated end joining (HMEJ)

The first report of CRISPR–Cas9 working in *Y. lipolytica* was published by Schwartz et al. [52]. In this work, the expression of the sgRNA was identified as limiting CRISPR–Cas9 activity. A codon optimized *S. pyrogen* Cas9 gene was fused with the SV40 nuclear localization signal and was expressed from the strong UAS1B8-TEF promoter. Initial tests with RNA Polymerase II TEF promoter, RNA Polymerase III SNR52 promoter and glycine tRNA resulted in moderate indel efficiency in the range of 30–40%. This is in contrast to *Saccharomyces cerevisiae* where SNR52-based promoters allow for high Cas9 cutting efficiencies. Improved expression of sgRNA and improved Cas9 editing efficiency was achieved though hybrid RNA Polymerase III promoters (RPR1, SCR1, and SNR52) fused to a glycine tRNA (Fig. 1a) to recruit *Y. lipolytica*’s native RNA processing machinery. Of the constructs tested, SCR1-tRNAgly produced the most frequent indels, suggesting the most effective and frequent Cas9 cutting activity. Interestingly, high expression and high editing efficiency did not correlate one-to-one suggesting that gRNA mutation and processing were also influential. The SCR1-tRNAgly RNA promoter and a cloning site for easy sgRNA insertion was placed on the Cas9 expression plasmid and called pCRISPRyl (Addgene #70007).

Shortly after, another report of CRISPR–Cas9 was published that demonstrated multiplexed gene knockouts [53]. The disruption plasmid consists of codon-optimized *S. pyrogens* Cas9 driven by the *Y. lipolytica* strong TEF-intron promoter and a sgRNA cassette with sgRNAs flanked by self-cleaving hammerhead ribozyme and hepatitis delta virus ribozyme sequences. By placing up to three such sgRNA cassettes in a single plasmid denoted pCAS1yl (Addgene #73226), three simultaneous disruptions were achieved with an efficiency of ~ 19%.

Type II CRISPR–Cas9 systems often rely on the host organism’s RNA processing machinery for production of mature guide RNA transcripts, however Morse et al. [54] have developed an orthogonal T7 polymerase-based system for guide RNA expression. The system consists of a T7 polymerase (T7 pol) with SV40 nuclear localization tag driven by a strong constitutive promoter and a T7 phi9 promoter driving the guide RNA expression. This design confers several advantages: independent tuning of sgRNA expression level via T7 promoter and/or T7 pol variation choice, portability between multiple hosts (e.g., *S. cerevisiae*, *K. lactis* and *Y. lipolytica*), and independence from native RNA processing machinery. In the process of developing this tool, Morse and colleagues provided additional evidence supporting previously observed guide RNA error tolerance via addition of 5′ guanines. Previous work showed improved tolerance at the cost of reduced performance when two 5′ guanines were present, however the addition of a three-guanine motif (GGG) recovered cutting efficiency while retaining error tolerance.

In order to determine the factors influencing CRISPR–Cas9 efficiency in *Y. lipolytica*, Borsenberger et al. [55] conducted a series of experiments with a strain of *Y. lipolytica* containing an integrated RedStar2 red fluorescent protein reporter. First, by varying the strength of the promoter on a codon-optimized Cas9, they demonstrated that increasing levels of Cas9 do not result in improved indel formation. On the contrary, they hypothesize overexpression of this protein can be toxic based on the increased death rate of Cas9-expressing cells. Unlike Cas9 expression, production of the sgRNA is the critical factor affecting the efficacy of CRISPR indel formation. An sgRNA directly fused to tRNA glycine without the aid of a spacer/linker, produces the most reliable cutting and subsequent indel formation compared to those attached with either a 4 or 9 base linker consensus sequence. Time course data indicated that cutting and indel formation occur relatively rapidly (< 30 h post transformation). The sgRNA was found to undergo an active production–editing–degradation cycle, supporting the need for sufficiently strong sgRNA complex expression. Finally, by exploiting the rapid cutting and repair action, transient ssDNA oligonucleotides were genomically integrated at CRISPR cut sites with a 16% efficiency.

Wanting to find safe landing sites for heterologous genes, a CRISPR–Cas9 system for markerless integration of a cassette into any one of five characterized genomic sites was developed [27]. The two-plasmid system works by expressing a codon-optimized, nuclear-targeted Cas9 and a corresponding sgRNA driven by the SCR1-tRNAgly synthetic promoter on one plasmid (Addgene #84608-12) while a second co-transformed plasmid contains a cassette with the integration construct flanked by 1 kbp upstream and downstream homologies to the integration site (Addgene #84613-17). Integration sites include genes for alternative substrate metabolism (MFE, AXP and XPR3) and pseudogenes (A08 and D17). These five sites chosen were non-essential and performed consistently in both exponential and stationary growth phases. By exploiting the enhanced need for repair near the double stranded break (DSB), including homologous recombination (HR), heterologous sequences thousands of base pairs in length can be inserted to the target site with efficiencies between 48 and 69%, dramatically reducing the need for a selection marker. To demonstrate the usefulness of markerless integration, a lycopene pathway was rapidly assembled achieving 8.6-fold improvement in lycopene titer compared to the base strain. It is interesting to note that 75% of the integration sites tested failed to produce appreciable integration, due to either poor sgRNA design, recalcitrance to HR, or some combination thereof.

CRISPR–Cas9 systems often achieve gene knockouts via indels resulting in frameshift mutation, which can lead to the production of short nonsense proteins of unknown function. In an effort to alleviate potential problems caused by such proteins, Gao et al. [56] demonstrates a CRISPR-based full gene excision knockout strategy. The system exploits *Y. lipolytica*’s non-homologous end-joining (NHEJ) to make the repair of the two simultaneous DSBs. By placing two sgRNA cassettes targeting immediately upstream and downstream of the gene of interest, respectively, full genes as long as ~ 3.5 kbp were able to be removed with an efficiency of 14–33%. In addition to gene excision, it is also possible to observe single or double indel formation at the target sites. Even though gene excision is not as efficient as indel formation, it is significantly easier to screen by colony PCR compared to indel screens, such as the surveyor assay.

Additionally, they demonstrated that this dual cleavage CRISPR–Cas9 method can be used as a means of targeted integration via the inclusion of a second “donor” plasmid containing target site homology and the desired integration product. When such a donor plasmid is provided, insertion and repair can proceed in one of two ways: HR or homology-mediated end-joining (HMEJ). The former method uses the donor plasmid as a template for repair while the latter liberates the insertion cassette and integrates it directly into the genome (Fig. 1b). The latter method, HMEJ, was shown to be more than twice as efficient (~ 37%) as HR (~ 16%) for integration of a desired sequence. Furthermore, in the HMEJ method, gene excision without integration dropped from ~ 15% down to less than 7% concomitant with increased integration efficiency. This was the first report of HMEJ for gene editing in any microorganism.

To facilitate the rapid engineering of *Y. lipolytica*, Holkenbrink et al. [57] developed a suite of genetic tools to enable easy integration and knockout of candidate genes from a series of pre-designed plasmids and oligos. The system, EasyCloneYALI (Addgene Kit 1000000140-141), consists of both markerless and marker-containing integration constructs using hygromycin, nourseothricin, or URA3. The markered constructs contain loxP site flanking their respective resistance genes to allow for marker recycling via the use of Cre recombinase. Integration can occur randomly or at one of 11 predetermined high-expression genomic loci. The sites chosen consistent of regions of approximately 5000 base pairs that contained no recognized open reading frames (ORFs), no known non-coding RNA elements, and were flanked by at least five highly-expressed ORFs. An integration cassette could be inserted into these sites by selection of the appropriate construct which contains 500 bp homologies upstream and downstream of the compatible Cas9 recognition site. Plasmids were constructed with common restriction sites and matching backbone homologies to allow for quick assembly. Knockouts are achieved by exploiting Cas9’s targeted cutting in combination with short oligos designed to exploit the yeast’s homologous recombination mechanism. High integration efficiency ranging from 30 to 80% was achieved for different sites in a Ku70 knockout background, resulting from the use of a non-replicative Cas9 plasmid.

Prior to the application of CRISPR–Cas9 for gene editing, Transcriptional Activator-Like Effector Nucleases (TALENs) were the preferred means of targeted gene and genome editing in yeast and mammalian systems. Rigouin et al. [58] reported the first and only use of TALENs in *Y. lipolytica* by mutating the ketoacyl synthase domain (I1220) on FAS1 to modify the fatty acid profile with for a biokerosene application. I1220 residue was mutated to all possible amino acids. The TALEN, cleavage site was centered around the I1220th residue. TALEN use led to 97% of NHEJ repair at FAS1 domain. Subsequently, 2 kb exogenous DNA was provided as a template for HR around the I1220 residue to mutate the isoleucine residue. Sequencing of colonies showed that TALEN was 40% efficient at HR.

## Modulation of gene expression in *Y. lipolytica*

CRISPR-inhibition (CRISPRi) was develop in *Y. lipolytica* by expressing a Cas9 with N10A and H840A mutated catalytic residues (dCas9) and a sgRNA targeted a specific gene for repression [59]. Several targets were chosen for CRISPRi repression to improve homologous recombination (KU70, KU80, DNL4, MIH1, ZDS1, STT4, SIN3, TUB1 and TUB4). The repression efficiency was increased by targeting sgRNA to the transcriptional start site (TSS) and TATA box. The TSS was identified through RNAseq data and TATA box was identified by searching for consensus TATA box 120 bp upstream of TSS. The repression efficiency was further increased by fusing either the KRAB domain or the Mxi1 repressor domain to dCas9. Fusion of Mxi1 to dCas9 provided the highest repression efficiency (Addgene #91248). The optimized CRISPRi tool was used to downregulated KU70 by 90% and KU80 by 83%. Integration of URA3 into different loci (PEX10, XDH, XLK and D17) showed an increase in percentage of HR compared to control strain. Not surprisingly, the KRAB domain failed to improve gene repression in yeast due to its non-conserved metazoan origin.

In a related work, CRISPRi was established by expressing dCpf1 or dCas9 to repress chromosomally integrated GFP [60]. dCpf1 produced 78% repression efficiency and dCas9 produced 89% repression efficiency. KRAB domain was fused to dCas9 and dCpf1 to increase repression efficiency. However, once again no improvement was observed for KRAB domain fusions. Experiments were conducted to establish the relationship between targeting site (template strand, non-template strand and promoter/coding region) and repression efficiency. Since no clear correlation was obtained between repression efficiency and targeting sites, multiple targeting sites (up to three targeting sites) were established through golden gate cloning. Up to 92% GFP repression efficiency was achieved through triple gRNA targeting (promoter, non-template strand and template strand) for dCas9 and 85% GFP repression efficiency was achieved through triple gRNA targeting for dCpf1. As a proof of concept, protodeoxy-violaceinic acid producing genes were simultaneously repressed through the optimized multiplex CRISPRi with 60–70% efficiency.

CRISPR activation (CRISPRa) of transcription was established by fusing transcriptional activation domain to dCas9 and tested through activation GFP expression [61]. Different activation domains (Gal4, VP16, VP64 and VPR) were tested for their impact on increasing the expression of GFP. VPR activation domain produced the highest expression compared to other domains. sgRNAs were targeted to different sites upstream of the coding sequence to increase the expression. The sgRNAs targeted upstream of the core promoter produced significant activation compared to those targeted to core promoter. The optimized CRISPRa system was used to upregulate two β-glucosidases (BGL1 and BGL2) that confer growth on cellobiose. Multiplexed activation of BGL1 and BGL2 led to 112-fold and 20-fold increase in expression of BGL1 and BGL2, respectively. Consumption of cellobiose demonstrated the impact of upregulation of BGL1 and BGL2.

## Future advances in gene editing and expression modulation in *Y. lipolytica*

The rapidly advancing gene and genome editing field will show itself in *Y. lipolytica* in the near future. Improved Cas9 variants with higher fidelity and alternative PAM sequence preference will be helpful for targeting different sequences throughout the genomes [62]. It remains to be seen if these variants will function the same way in *Y. lipolytica* as they function in mammalian cells. The implementation of Cas12a (CpfI) will likely make editing promoter regions easier due to its T-rich PAM sequence [63]. Base editor technology [64] has not yet been developed for *Y. lipolytica*; however, it would be a useful tool for making specific edits directly in the genome without the complication of a second donor DNA. Improving the rate of HR is also important for hastening strain engineering. This could be achieved overexpressing components of the HR machinery or downregulating NHEJ components. Another system that would be useful for strain engineering is the serine integrases, which mediate site-specific efficient integration [65]. Finally, epigenetic modifiers based on dCas9 fusions [66] are likely to become available for studying gene regulation and cryptic gene activation in *Y. lipolytica*.

## Genome scale engineering and functional genomics of *Y. lipolytica*

The power of gene editing tools is realized when they are applied at the genome scale. There are three recent works that have created genome scale libraries for the purpose of functional genomics studies and for strain engineering. This section discusses each approach and what was learned.

A functional piggyBac transpose system was developed through the expression of a hyperactive piggyBac transposon (hyPBase) from an episomal plasmid [67]. The piggyBac transposon is TTAA-specific and has several useful features such as propensity for inserting in transcriptional units, mobilizing large DNA sequencing, and lack of transposase toxicity. This system was validated through reactivation of GFP reporter and tryptophan prototrophy. The functional piggyBac system was used to create an insertional mutagenesis library which was screened for canavanine resistance, coloration through *ade* disruption and lipid accumulation by Nile Red stain. High lipid content resulted from the presence of insertion on the upstream of MHY1 gene and the low lipid content resulted from absence of DGA1 gene. This is consistent with the previous studies conducted in *Y. lipolytica*.

The authors have shown that the excision frequency and re-integration frequency of piggyBac transposase is similar for both chromosomally integrated GFP and plasmid integrated TRP genes. They measured the excision frequency by integrating a URA3 marker between GFP and TRP. Successful excision resulted in functional GFP and TRP quantified by flow cytometry and colony counts on SD-Trp media, respectively. To test for re-integration frequency, the sorted GFP positive cells and TRP positive cells were plated on uracil containing plates. Fourteen-fold more transformants were obtained compared to the control lacking piggyBac ITRs. Introduction of three mutations (R372A, K375A, D450N) into the hyPBase produced hyEXC (an excision/integration mutant). This hyEXC was further used to excise and recycle selection markers, although recombination was 1.7-fold lower compared to the popular Cre recombinase excision. While mutagenesis with the piggyBac system is limited to sequences containing TTAA, the piggyBac system can increase random integration rates of DNA into the genome of *Y. lipolytica*.

Functional genomics studies require systematic and genome-wide perturbations that can be rapidly correlated to a phenotype. Patterson et al. [68] enabled functional genomics in *Y. lipolytica* by developing a library over 534,000 independent Hermes transposon (HTn; Addgene 113332) random insertions throughout the genome. The Hermes transposon library was used to classifying genes as essential, low-confidence (LC) essential and non-essential for growth on glucose or glycerol by measuring the change in abundance of insertion mutants after growth. The results showed that almost 22% of the genes in *Y. lipolytica* were classified as essential, 9.3% were LC essential and 67.8% were non-essential. Comparison showed that 73.4% and 69.5% of the essential genes in *S. cerevisiae* and *S. pombe* were also essential in *Y. lipolytica*. This result indicates there are significant differences between model yeast and *Y. lipolytica* biochemistry. Comparing the essential genes for growth on glucose or glycerol indicates glycerol metabolism requires more genes, likely due to the lower energy content of glycerol. It was also noteworthy that four of the 21 TCA cycle-associated genes were classified as growth impairing but non-essential even though *Y. lipolytica* is an obligate aerobe. Importantly, the functional genomics data did not agree with predictions of gene essentiality from flux balance analysis of two genome scale metabolic models (GEMs), indicating the strong need for using functional genomics to validate and refine GEMs.

The Hermes transposon library was also used for isolating mutants with altered lipid metabolism on the basis of BODIPY fluorescence intensities. The top 1% of FACS sorted library was found to have altered lipid metabolism in a nitrogen-rich medium and higher total lipid content in a low-nitrogen media. In isolated clones, an insertion upstream of YALI1_F11261g (unknown function) and an insertion upstream of the vacuolar protease, PRB1 resulted in more lipid than the control strain.

Recently, the first CRISPR–Cas9 genome scale indel library was constructed for functional genomics and strain engineering in *Y. lipolytica* [69]. This library contains cells with single knockouts of nearly all genes in the genome. The library can be used to determine gene essentiality by growth in particular media, where the essential gene knockouts are selected against and therefore are less abundant after a period of outgrowth. The so-called fitness score (FS) was used to quantify the degree to which a particular gene knockout resulted in its lack of growth and therefore, loss of abundance from the library. The FS is calculated by taking log_2_ of the abundance of each individual sgRNA sequence in a Cas9-expressing strain after selection, normalized to the abundance of each individual sgRNA sequence in a Cas9-deficient strain (i.e., non-edited control library).

One of the main challenges of developing a CRISPR-based library screen for a non-model system is identification of active sgRNAs. The resulting strategy, therefore, uses six sgRNAs to target each open reading frame in the genome. Specific sgRNAs were designed to target first 300 base pairs in each gene so indels would likely lead to a premature stop codon. Assessment of the sgRNA activity was determined by a so-called cutting score (CS). The CS is calculated by taking log_2_ of the abundance of each individual sgRNA sequence in a Cas9-expressing KU70 knockout strain after selection, normalized to the abundance of each individual sgRNA sequence in a Cas9-deficient strain (i.e., non-edited control library). The KU70 knockout eliminates NHEJ repair making efficient cutting by a particular sgRNA-Cas9 complex a lethal phenotype.

A CRISPR–Cas9 library of single guide RNAs targeting each of the 7854 coding sequences with sixfold redundancy was designed, constructed and transformed into either WT *Y. lipolytica* PO1f, PO1f with an integrated Cas9 gene, or PO1f with an integrated Cas9 gene and KU70 knockout. Sequencing analysis showed that 97% of the designed sgRNAs were well represented in the library. The libraries were passaged every 2 days, resulting in weak selection by day two and significant selection by day 4. By this time, it became apparent that many sgRNAs did not cut efficiently and had high FS and CS scores (high scores mean little impact on fitness and weak cutting, respectively).

Several features of non-cutting and poorly cutting sgRNAs were analyzed to determine modes of failure of sgRNA targeted cutting. The presence of a polyT motif in a sgRNA correlated with reduced CRISPR–Cas9 activity, while the RNA secondary structure did not significantly impact activity. The sgRNAs at the ends of the chromosome were largely inactive suggesting lower CRISPR–Cas9 activity could be caused by chromatin structure. Weak correlation between nucleosome occupancy and sgRNA activity was also observed. Combined these results confirm that sgRNA design algorithms are as good as the data on which they are trained.

Data analysis of FS and CS indicate that poor cutting sgRNAs are responsible for producing false negatives, which complicate data interpretation. When the naïve library was used to compare essential and non-essential genes, the difference between their FS distributions was not significant. It is well known that ACT1, MYO1, FOL2 are essential for eukaryotic cell growth but in the naïve library they had similar FS compared to non-essential genes. When the validated library for sgRNAs was built by filtering out the low CS data, there was a significant difference between the distributions of essential and non-essential genes. The authors concluded that the validated library generates more accurate data because it consists of only highly active sgRNAs compared to the naïve library.

Using the validated library 1377 (17.5% of the genome) was classified as essential. This is similar to the number of essential genes and the percentage of the genome that is essential in *S. cerevisiae* and *S. pombe*. The essential genes in *Y. lipolytica* were compared to homologous essential genes in *S. cerevisiae*. A total of 960 homologs were identified and 480 were essential for both organisms. There were 416 genes that were essential in *Y. lipolytica* but not in *S. cerevisiae*. They were also compared to essential genes in *S. pombe*. Of 198 *Y. lipolytica* genes that had homologs in *S. pombe*, 111 were essential. Interestingly, the transposon library experiment performed by Patterson et al. [68], identified 586 more essential genes. Only 67% of the essential genes identified with the validated CRISPR–Cas9 library were also classified as essential by the transposon screen. It remains unclear which method is more accurate and if either method significantly influences gene expression outside of the target gene.

This work also demonstrated the usefulness of the CRISPR Cas9 library for novel phenotype screening. The first screen was based on canavanine resistance which led to the identification of sgRNAs of excellent cutters targeting CAN1 as expected. The second screen was for increased lipid content in the cells which was evaluated using red fluorescent lipid dye FACS. Results from this experiment revealed expected and unexpected targets, highlighting the usefulness of genome-scale library screening for identifying non-obvious targets for strain engineering.

Embedded in the data are some of the system limitations. For example, there was noticeable burden associated with expression of Cas9, which creates an additional selection pressure that may influence the interpretation of gene essentiality results. Another shortcoming of CRISPR-Csa9 knockout libraries is that the desired phenotype might only be accessible through gene overexpression.

## Future advances in functional genomics and genome scale engineering

The extension of knockout library screening for gene essentiality in alternative substrates is a straightforward application of CRISPR–Cas9. Similarly, extending the CRISPR–Cas9 knockout library to screening for other useful production phenotypes is expected. Combining the libraries with reporters for secreted products, for example [70], could lead to new insights for metabolic engineering. The most obvious next step in genome scale engineering is the extension of CRISPRa [61] to a genome-scale library. The remaining barriers to genome scale implementation relate to the scientific challenge of identifying the optimal location for targeting dCas9-VPR fusions. Without this understanding one would need to create a significantly larger library than we currently have the capacity to make. A wrinkle in this theme would be the use of Cas12a to more easily target the activation complex to the promoter region. A less obvious advance that is needed would be a high throughout means of functional annotation of genes of unknown function. Much of the genome annotation in *Y. lipolytica* is based off of homology to genes in other organisms that likewise do not have definitive function. Assigning function to the genome with some degree of confidence cannot be achieved by homology alone. Rapid methods for not only interrogating the essentiality of each gene but also the localization and interaction partners will help create a complete picture of what each gene does. This type of deep understanding would improve genome scale models and make strain engineering more of a science than art.

## Conclusions

In order to fully realize *Y. lipolytica*’s capacity for industrial scale production it is essential to continue developing gene and genome editing tools that can be applied to strain engineering and functional genomics. Expected advances in these tools for mammalian cells will likely impact *Y. lipolytica* tool development. Key advances in genome-scale libraries for gene knockout and activation and other functional genomics tools will be brought to bear towards developing a more accurate picture of cellular metabolism and regulation. It is ultimately this deep knowledge that will unlock the full potential of this non-conventional yeast as an industrial host for production of bio-based products.

## Data Availability

The datasets supporting the conclusions of this article are included within the article or the referenced literature.

## Abbreviations

T7 pol: T7 polymerase
DSB: double stranded break
HR: homologous recombination
NHEJ: non-homologous end-joining
HMEJ: homology-mediated end-joining
ORFs: open reading frames
TALENs: Transcriptional Activator-Like Effector Nucleases
CRISPRi: CRISPR-inhibition
TSS: transcriptional start site
CRISPRa: CRISPR activation
hyPBase: hyperactive piggyBac transposon
HTn: Hermes transposon
LC: low-confidence
GEMs: genome scale metabolic models
FS: fitness score
CS: cutting score
